# Current pathways model for hall thruster plumes in ground-based vacuum test facilities: measurements and observations

**DOI:** 10.1007/s44205-024-00097-8

**Published:** 2024-12-30

**Authors:** David R. Jovel, Janice D. Cabrera, Mitchell L. R. Walker

**Affiliations:** 1 Georgia Institute of Technology, Atlanta, GA 30332 USA; 2 School of Aerospace Engineering, Atlanta, USA

**Keywords:** Electrical facility effects, Hall effect thruster, Plasma plume, Plume facility interaction

## Abstract

A previous companion paper introduced a current pathways model that represents the electrical coupling between the Hall effect thruster (HET) and the ground-based vacuum test facility operational environment. In this work, we operated a 7-kW class HET at 4.5 kW, 15 A and 6 kW, 20 A on krypton to quantify aspects of the current pathways model to characterize the role metal vacuum chambers play in the thruster’s discharge circuit as a function of discharge current. During HET operation, far-field ion and electron saturation currents at 47 near-facility wall locations were measured using an array of 5.08 cm diameter, stainless-steel planar electrodes. In addition, the plasma properties at three distinct locations within the facility, 25 cm from the facility wall, were obtained using Langmuir probes. Experimental results show that thruster beam ions do not readily neutralize with cathode electrons and instead neutralize with the free electrons provided by the metallic chamber wall. In addition, significant charge-exchange (CEX) ion current was measured in the background plasma environment and constituted about 23% of the total ion current measured by the planar electrode array. Thus, the metallic vacuum chamber surfaces facilitate charge neutralization for both ion populations. Additionally, the plasma environment near the facility walls was characterized to be non-uniform with an estimated plasma sheath capacitance ranging between 0.45 *µ*F and 1.79 *µ*F. Further analysis shows that the plasma sheath at the facility wall behaves like a parallel RC circuit, potentially concealing the thruster’s AC characteristics. Inherent plasma oscillations give rise to inductive effects with inductances that varied between 76.5 nH and 101.4 nH. Hence, the dynamic characteristics of the HET’s discharge are influenced by the capacitive and inductive effects introduced by the vacuum test facility operational environment.

## Introduction

The performance and stability of Hall effect thrusters (HETs) depend on their local operating environment. Ground-based vacuum test facilities used for HET performance characterization cannot replicate many aspects of the real operating environment in space. For instance, vacuum test facilities are comprised of electrically conductive metal surfaces, have finite volume, and can only maintain operational pressures that are four orders of magnitude higher than what is measured in the space environment [1]. The difference between these two operating environments has motivated research in characterizing the effects that test facilities have on the performance of the thruster within the electric propulsion (EP) community. These effects, formally called facility effects, can be divided into three categories: (1) contamination, (2) pressure, and (3) electrical. Of the three categories, electrical facility effects is a relatively new research area and will be the focus of this article.

Electrical facility effects research investigates how the thruster’s electrical configuration and the resulting coupling between the emitted plasma plume and the surrounding vacuum chamber affect the thruster’s performance and stability. For clarity, we expand on the terms *electrical configuration* and *coupling*. First, we define the thruster’s ‘*electrical configuration*’ to be the wiring between the thruster body relative to the cathode body and the grounded test facility and how, together, they complete the discharge electrical circuit. The appropriate thruster electrical configuration should be representative of the wiring aboard the spacecraft to replicate flight-like conditions and remains an open area of research within the community. Second, ‘*coupling*’ is defined to be the exchange of electric charge between the HET plasma plume and the surrounding vacuum test facility walls. Such an interaction must occur at the plasma-facility wall interface because both the plasma plume and the metallic vacuum chamber walls are electrically conductive media. Thus, it is reasonable to deduce that vacuum test facilities may influence the electrical *I-V* characteristics of HETs during ground-based test campaigns. Despite this, the role vacuum chambers serve in accommodating HET plume ion and electron currents has not been adequately studied nor quantified for the community. Given this background it may seem that electrical facility effects research is underdeveloped. However, some significant work has already been accomplished in this area and is briefly summarized next.

The first studies characterizing the effect of the HET’s electrical configuration on its performance and dynamic behavior were conducted by Peterson et al. in [2]. In this work, Peterson et al. demonstrated that the HET’s thrust and dynamic behavior, in terms of peak-to-peak and root-mean-square discharge current measurements, varied for three different electrical configurations. In addition, Peterson also showed that thruster body’s electrical conductivity also affects the measured discharge current oscillations. Next, Walker et al. were the first to provide evidence of electrical coupling between the HET plasma plume and the surrounding vacuum chamber in [3]. Walker conducted multiple experiments in which the thruster body and/or conductive surfaces inside the test facility were controlled to manipulate the electron current pathways present during HET operation. Specifically, Walker quantified the effect of changing one of the following three variables on HET electron current pathways: (1) the relative position of an externally-mounted cathode with respect to the thruster centerline and chamber wall, (2) the thruster body’s electrical configuration with respect to facility ground, and (3) the voltage of large electrodes exposed to the HET plume relative to facility ground. More recently, Jovel et al. developed a theoretical framework that can be used to qualitatively understand how the local operating environment affects the dominant ion and electron current pathways during HET operation in [1]. Jovel established that vacuum test facilities provide two additional current pathways that help satisfy the net charge neutral volume boundary condition compared to the space operational environment. These additional current pathways were shown to be associated with the elevated pressures maintained inside vacuum chambers and their electrically conductive walls. The work presented in this paper is an extension of [1] in that we aim to quantify aspects of the current pathways model presented.

The primary goal of this work is to quantify the degree of electrical coupling between the HET plasma plume and the vacuum test facility for a grounded thruster body electrical configuration. To achieve this goal, we will aim to satisfy the following objectives: (1) quantify the amount of ions and electrons fluxing to the facility wall during thruster operation, (2) show that the magnitude of ions and electrons fluxing to the facility walls increases as the HET’s discharge current level increases, and (3) show that the test facility introduces capacitive and inductive effects that ultimately influence the dynamic behavior of HETs. Our approach will be to leverage the current pathways model developed by Jovel et al. in [1] to identify key interfaces where ions and electrons generated by the HET interact with the vacuum test facility. For sufficient spatial resolution, the plasma environment contained within the facility is decomposed into 47 near-facility wall locations, covering a 0° to 180° line-of-sight to the HET exit plane, each with a 5.1-cm circular electrode that can be electrically biased to collect local *I-V* characteristics. We will show that both ions and electrons are present, and vary in magnitude, across all 47 near-facility wall locations establishing a spatially non-uniform plasma environment. As a result, we provide evidence that HET beam ions are less likely to recombine with cathode electrons in the plume and are instead effectively neutralized by the metal vacuum chamber walls. Moreover, these measurements confirm the presence of charge-exchange (CEX) ions and their role in redistributing the ion current throughout the facility. Furthermore, we will use Langmuir probe data to quantify the plasma-facility wall sheath capacitance and plasma oscillation inductance and their effect on the dynamic discharge characteristics of the HET. Lastly, we will show that the electrical coupling between the HET plasma and the test facility increases as the HET’s discharge current magnitude increases.

The organization of this manuscript is as follows. In section II, we review the current pathways model presented in [1] and the assumptions used in its development. The objective is to familiarize the reader with the theoretical framework used to guide this work. In section III, we provide an overview of the experimental setup consisting of the HET test article, test matrix, vacuum test facility, and plasma diagnostics. In section IV, we present the experimental data comprised of ion and electron saturation currents at 47 near-facility wall locations, Langmuir probe data at three distinct locations within the facility, and Faraday sweeps to quantify the HET’s ion beam current and divergence angle. The data presented in section IV is used to quantify the role metal vacuum chambers play in maintaining a charge-neutral environment. In section V, we examine the results and discuss their implications in characterizing the dynamic behavior of HETs. We conclude by summarizing the experimental data that confirms that the HET plasma plume electrically couples to, and is be affected by, the ground-based vacuum test facility’s pressure and electrically conductive material properties. We further discuss the implications of this coupling for higher power HETs.

## HET discharge-facility current pathways model

**Fig. 1:**
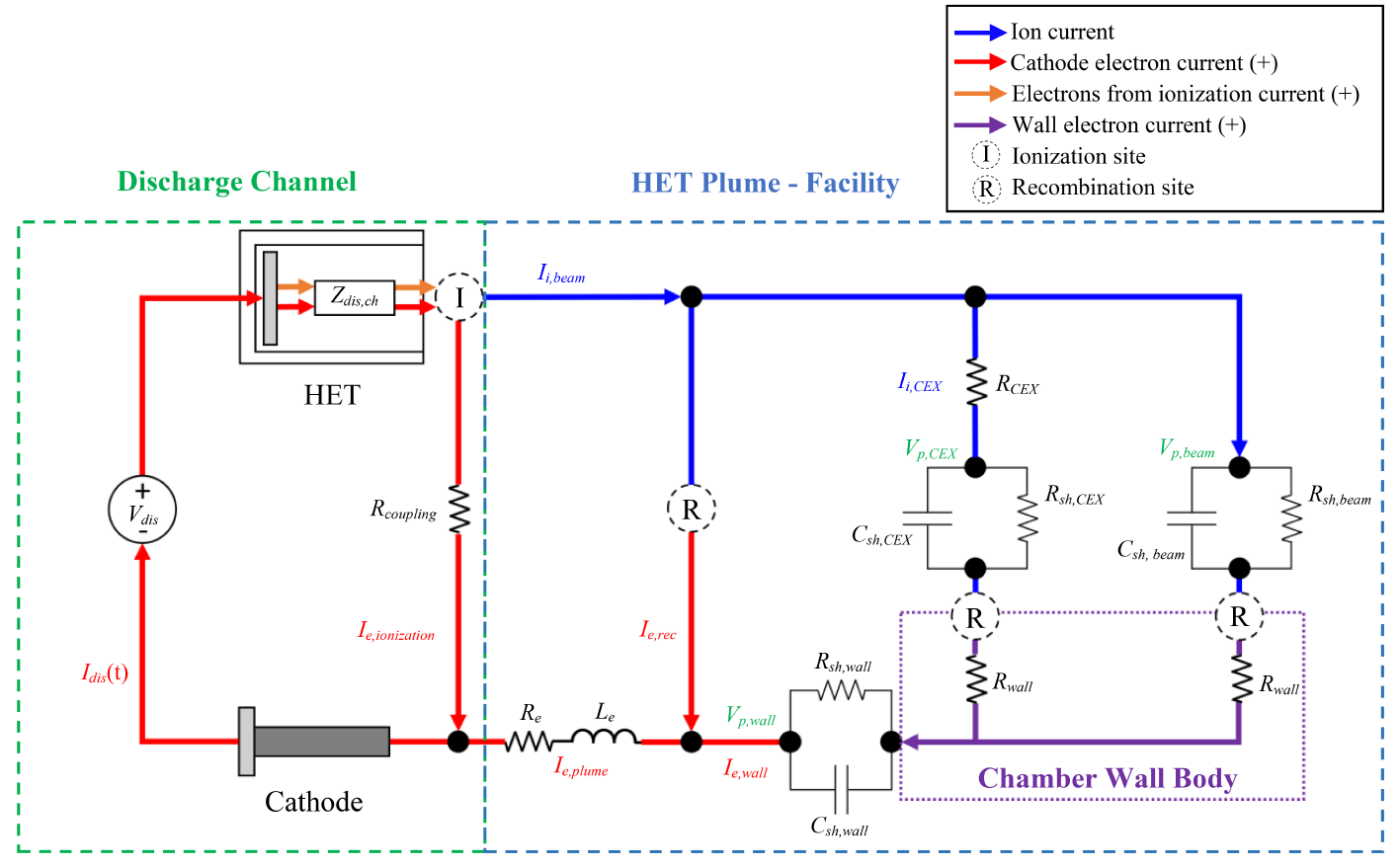
HET Discharge-Facility Current Pathways Model

In this section, we provide a thorough overview of the current pathways model developed by Jovel et al. in [1]. The objective of the current pathways model is to represent the electrical coupling between the HET discharge and the vacuum test facility using fundamental electrical circuit elements. Figure 1 shows the model with only one cross section of the HET’s discharge channel depicted for clarity. The model will be used to understand how plume-facility interfaces can potentially influence the dynamic characteristics of the HET discharge.

### Approach & assumptions

Our approach in developing a representative HET discharge-facility current pathways model is as follows. The 3D, non-uniform plasma encapsulated by the vacuum facility is decomposed into effective current pathways that physically interact with the thruster discharge and the vacuum test facility walls. Each pathway is a bulk, macroscopic representation of various spatiotemporal, microscopic physical processes responsible for sourcing, sinking, or exchanging charge within a given operational environment. This work has identified seven charge-related processes that occur simultaneously and throughout the facility during HET operation: (1) cathode electrons conducting to the discharge channel and colliding with neutrals producing beam ions, (2) beam ions accelerating downstream of the HET exit plane and colliding with background neutrals producing CEX ions at elevated pressures, (3) beam ions recombining with cathode electrons within the plume, (4) CEX ions recombining with cathode electrons within the plasma volume, (5) beam ions fluxing to the facility walls and recombining with chamber wall electrons, (6) CEX ions fluxing to the facility walls and recombining with chamber wall electrons, and (7) cathode electrons fluxing to the facility wall to replenish the deficit of facility wall electrons that recombine with all ion species elsewhere. Each current pathway is represented as a circuit branch consisting of equivalent resistors, *R’s*, capacitors, *C’s*, and/or inductors, *L’s*, to capture the physics governing its interaction with the local operational environment. Moreover, each current pathway will be depicted as either blue (positive charge) or red (negative charge) to distinguish between ions and electron species. Furthermore, all current pathways are interconnected by effective electrical nodes to form a network akin to an electrical circuit. The model is closed by imposing a net charge neutral control volume boundary condition between all the ion and electron pathways mapped as the HET plume couples to the vacuum test facility.

The construction of the 2D, circuit-like network as shown in Fig. 1 is as follows. First, we use operational experience to divide the current pathways into two types: (1) current for sustaining the HET discharge during operation as registered by the DC discharge power supply and (2) all other currents related to maintaining a net charge neutral operational environment. Second, we arrange the current pathways in the order that they occur, starting from the main HET discharge, with respect to the space operational environment. This is because we aim to have an adaptable model that can be modified to identify the current pathways for flight-like conditions. As the operational environment changes, additional current pathways can be appended to the existing model to capture new charge-related processes unique to that operational environment as done for a facility in this study. Third, we place additional current pathways in parallel to depict the various branches in which charge is distributed to maintain a net charge neutral operational environment. Fourth, we use *R*’s to represent momentum loss of charge carriers, *C*’s to represent plasma sheath capacitance at plasma-wall interfaces, and *L*’s to represent inertial displacement of electrons due to an oscillating electric field in the plume. Their individual placement throughout the model is discussed in more detail in the following subsection.

The assumptions in the development of this model are enumerated next. First, we assume that the cathode is the only electron source in this model, and secondary electron emissions from chamber surfaces are neglected. Second, the plasma environment inside the test facility is a cold plasma, consisting of singly-charged ions, propellant neutrals, and Maxwellian electrons. Residual atmospheric constituents contained within the facility volume or sputtered materials are ignored. Third, we assume that the 3D electromagnetic phenomena occurring throughout the facility can be spatially confined to discrete *R*, *C*, and *L* components at effective node locations. In doing so, we develop a lumped-parameter network model [4]. Lastly, no net charge is allowed to accumulate on any of the components in the circuit. This assumption guarantees that the HET plume – facility current pathways segment of the model remains net charge neutral. Given this background, we discuss the HET Discharge – Facility Current Pathways model next.

### Model description

The current pathways model is divided into discharge channel processes and HET plume-facility processes as indicated by the two dashed boxes in Fig. 1. The DC power supply is connected to the anode and cathode electrodes, as shown, and is the only energy source for the model. The “Discharge Channel” branch of the model conducts the discharge current, *I*_*dis*_, at the fixed DC discharge voltage, *V*_*dis*_. We establish that *I*_*dis*_ is a time-varying parameter and is regarded as an AC current with a mean DC value defined by the nominal discharge operating point of the thruster. The “HET Plume - Facility” segment encompasses all the ion and electron current pathways required to satisfy plume neutralization and is the focus of this work. The positive current flow convention is adopted to remain consistent with circuit theory in electrical engineering. For locations where ion-electron recombination events occur, we use dashed circles with *R* throughout the circuit, indicating a current sink. At the same time, neutral-electron ionization inside the thruster discharge channel is denoted with *I* and represents a charge source. In the following subsection, we thoroughly examine the HET Plume-Facility current pathways as they served as the principal guide for this work. For insight on the “Discharge Channel” segment and other model details, the reader is encouraged to read [1].

#### HET plume-facility current pathways

The current pathways to the right of the Discharge Channel branch all comprise the thruster plume neutralization pathways inside ground-based vacuum chambers. The ion beam current originating in the discharge channel, *I*_*i, beam*_, is depicted in blue and runs along the topside of the circuit whereas the electron current for plume neutralization, *I*_*e, plume*_, is shown in red at the bottom. *I*_*i, beam*_ is divided into three branches that represent charge sink or exchange processes: (1) beam ion-cathode electron recombination events within the plume, (2) charge exchange with residual neutrals within the facility volume, *I*_*i, CEX*_, or (3) beam ions recombining with facility wall electrons. The first branch represents the charge neutralization process that occurs in the space operational environment. The CEX-ion and beam-ion neutralization branches at the facility wall embody the additional current pathways provided by ground-based vacuum chambers. To neutralize the three ion current pathways delineated, we decompose *I*_*e, plume*_ into *I*_*e, rec*_ and *I*_*e, wall*_. *I*_*e, rec*_ is the electron current required to neutralize the HET plume and the only pathway for neutralization in the space operational environment. In vacuum test facilities however, CEX and beam ions are more likely to recombine with chamber wall electrons since the mean free path for ion-electron recombination is > 5 km [1]. Therefore, *I*_*e, wall*_ is the electron current pathway to nearby facility surfaces required to replenish the chamber wall body with electrons to maintain a net charge neutral control volume. Lastly, the bottom right portion of the model shows the chamber wall’s role in facilitating charge neutralization. The placement of the chamber body is between *I*_*e, wall*_ and the CEX ion and beam ion pathways to depict its physical role in accommodating overall charge neutralization. The placement of *R*’s, *C*’s, and *L*’s throughout the model and their physical meaning is discussed in the subsequent sections.

#### Effective inductance of electrons in an oscillating plume

An oscillating E-field of a certain frequency will cause the electron conductivity to vary at the same frequency exhibiting an effective inductance, *L*_*e*_. The 1D electron conductivity provided in Eq. 1 can be separated into a real component that is a function of the total electron collision frequency, $\:{\nu\:}_{e,tot}$, and an imaginary component that is a function of the oscillating frequency, $\:\omega\:$ [5]. Comparing the imaginary term in the electron conductivity to the definition of inductive reactance provides us with an equivalent expression for *L*_*e*_ and given by Eq. 2. *L*_*e*_ is a function of the electron number density, *n*_*e*_, an effective length, *l*_*eff*_, and an effective area, *A*_*eff*_.

1 $$\:{\sigma\:}_{e}\left(\omega\:\right)=\frac{{e}^{2}{n}_{e}}{{m}_{e}\left({\nu\:}_{e,tot}+j\omega\:\right)}$$

2 $$\:{L}_{e}=\frac{{m}_{e}{l}_{eff}}{{e}^{2}{n}_{e}{A}_{eff}}$$

Selection of *l*_*eff*_ depends on the operational environment and can be 1’s of m’s for vacuum chambers or 100’s of m’s for the space environment. We assume that the electrons affected by the oscillating E-fields are all contained within a collimated HET plume. Thus, *A*_*eff*_ is defined to be the cross-sectional area of the HET based on its outer channel diameter and the same for both operational environments. For a vacuum chamber with an effective length of 6 m and a measured *n*_*e*_ of 10^16^ m^-3^ one-meter downstream of the HET exit plane, typical values for *L*_*e*_ are estimated to be 300 nH. The magnitude of the inductive reactance, given by the product of $\:\omega\:\:$ and *L*_*e*_, can achieve values in the 100’s of mΩ’s for oscillating frequencies on the order of kHz.

The inductance of electrons due to an oscillating plume is represented by a series resistor-inductor branch at the bottom of the model on the *I*_*e, plume*_ electron current pathway as shown in Fig. 1. The resistor represents the momentum scattering of electrons due to intermediate collisions with plasma constituents whereas the inductor captures the momentum displacement of the electrons given the time-varying E-field. The placement of this *RL* branch as shown ensures that the electron current emitted by the cathode for plume neutralization, *I*_*e, plume*_, must experience this inductive effect prior to recombining with ions in the plume or replenishing the electron deficit at the facility walls.

#### Ion-electron recombination pathway in the plume

The second branch depicts the ion and electron currents associated with recombination events within the HET plume. Expressing this charge-neutralization process with fundamental electronic components remains a challenge and is represented as a charge sink for this model. Regardless, we want to include a separate current branch dedicated to ion-electron recombination events in the plume as it is the only neutralization process in the space operational environment.

#### Beam-CEX ion resistance

The third pathway represents the generation and neutralization of CEX ions due to elevated facility pressures. In Fig. 1, a fraction of the ion beam current is diverted to CEX ion current via collisional events. Since we are tracking the momentum of charged particles throughout the model, a 300-eV beam ion exchanging charge with a slow-moving neutral atom appears to have lost much of its initial momentum in one direction. We represent this momentum loss by *R*_*CEX*_, and is provided by Eq. 3. For convenience, we have expressed *R*_*CEX*_ in terms of measurable inputs such as facility operational pressure, *P*_*op*_, chamber wall temperature, *T*_*w*_, mean CEX collision rate coefficient, $\:〈{\text{v}}_{i}{Q}_{CEX}〉$, and plume-facility geometry lengths *l*_*eff*_ and *A*_*eff*_.

3 $${R_{CEX}} = \frac{{{m_i}{P_{op}}\left\langle {{{\text{v}}_i}{Q_{CEX}}} \right\rangle {l_{eff}}}}{{{e^2}{n_i}{k_b}{T_w}{A_{eff}}}}$$

For this resistance, the choice for *l*_*eff*_ and *A*_*eff*_ are based on an effective location within the ion beam divergence cone, with half-angle *θ*_*div*_, at which CEX is assumed to occur. For example, *l*_*eff*_ can be the axial length between the thruster exit plane and the downstream facility wall, while *A*_*eff*_ can be the area of a circular cross-section of the expanding ion beam at *l*_*eff*_/2. Consider a HET accelerating 300-eV krypton ions with *θ*_*div*_ of 25° and *P*_*op*_ = 1 × 10^-5^ Torr-Kr, *T*_*w*_ = 300 K, *n*_*i*_ = 10^16^ m^-3^. Moreover, we reference [6] to obtain *Q*_*CEX*_ 4.4 × 10^–19^ m^2^ for 300-eV krypton ions. If the expanding ion beam has an axial length of 6 m, then the *R*_*CEX*_ is estimated to be 2 Ω.

This branch is placed parallel to the other branches as this process occurs simultaneously to all others depicted. Physically, the CEX branch is intended to represent the plasma environment within the facility that is not contained within the HET plume. We call this plasma the background plasma to distinguish from the more energetic plume plasma emitted by the HET.

#### Plasma sheath capacitance at the walls

A plasma sheath exists between the plasma medium and the facility walls to accommodate the local voltage boundary conditions [7]. The plasma sheath is a continuous, non-uniform 3D structure because the plasma contained within the facility is also a continuous, non-uniform 3D medium. To simplify this, we divide the plasma environment into two distinct regions. The first region is the high-energy HET plume plasma, consisting of beam ions and cathode electrons, contained within a cone defined by *θ*_*div*_, and the second region is the background plasma, consisting of only CEX ions and cathode electrons. The interface between the two plasma regions and the facility wall is represented by an effective plasma sheath structure. The plasma sheath structure is modeled as a capacitor and resistor in parallel with the DC capacitance given by Chen in [8]. For completeness, the sheath capacitance, *C*_*sh*_, appropriated from Chen, is provided below in Eq. 4 where *T*_*e*_ is given in eV. The sheath resistance, *R*_*sh*_, is governed by the conductivity of electrons fluxing through a 10 Debye length thick sheath and given by Eq. 5.

4 $$\:{C}_{sh}=\frac{{A}_{sh}}{2.38}{\left(\frac{{\epsilon\:}_{o}{n}_{e}e}{{T}_{e}}\right)}^{1/2\:}{\left(\frac{\left({V}_{p}-{V}_{w}\right)}{{T}_{e}}\right)}^{-0.75}$$

5 $$\:{R}_{sh}=\frac{{m}_{e}{\nu\:}_{e,tot}10{\lambda\:}_{D}}{{e}^{2}{n}_{e}{A}_{sh}}$$

The placement of the plasma sheath structures is between their respective ion current pathways and the chamber wall body segment of the model. Note that electrons also flow through both plasma sheath structures shown. For clarity, we use one equivalent plasma sheath structure on the electron pathway before the chamber wall segment to represent the conduction of electrons in the two other plasma sheaths.

It is clear from Eqs. 4 and 5 that to estimate the capacitance and resistance of the plasma sheath, the local plasma properties are required. Langmuir probes can be used to quantify the plasma properties for the background plasma and the plume plasma regions. The physical boundary distinguishing the two plasma regions is provided by the intersection of the diverging ion beam with the facility wall enclosure. The sheath area, *A*_*sh*_, is the wetted facility surface area incident to the plasma region of interest. The sheath capacitance for both plasma regions is on the order of *µ*F, and the sheath resistance is negligible and below 5 *µ*Ω.

Together, these branches provide a framework for understanding the numerous plasma processes in a vacuum test facility. The model’s usefulness can further be enhanced by experimental data that provides orders of magnitude estimates of the *R’s*, *L’s*, and *C’s* present in the model. For example, measuring the ion flux to electrodes placed throughout the facility gives a fraction of the total ion beam current associated with the two ion current pathways interacting with the facility wall. Hence, we quantify the degree of electrical coupling between the plasma environment and the facility with the plasma parameters gathered from experimental data.

## Experiment overview

In this section, we provide an overview of the experiment and provide details regarding the thruster, vacuum test facility, and diagnostics employed. The focus of this section will be on the witness plate array diagnostic used to measure the ion and electron saturation currents at 47 near-facility wall locations, the Langmuir probes used to ascertain the plasma properties at three locations within the facility, and the Faraday probe used to determine ion beam current as well as beam divergence half-angle.

### Thruster

**Fig. 2:**
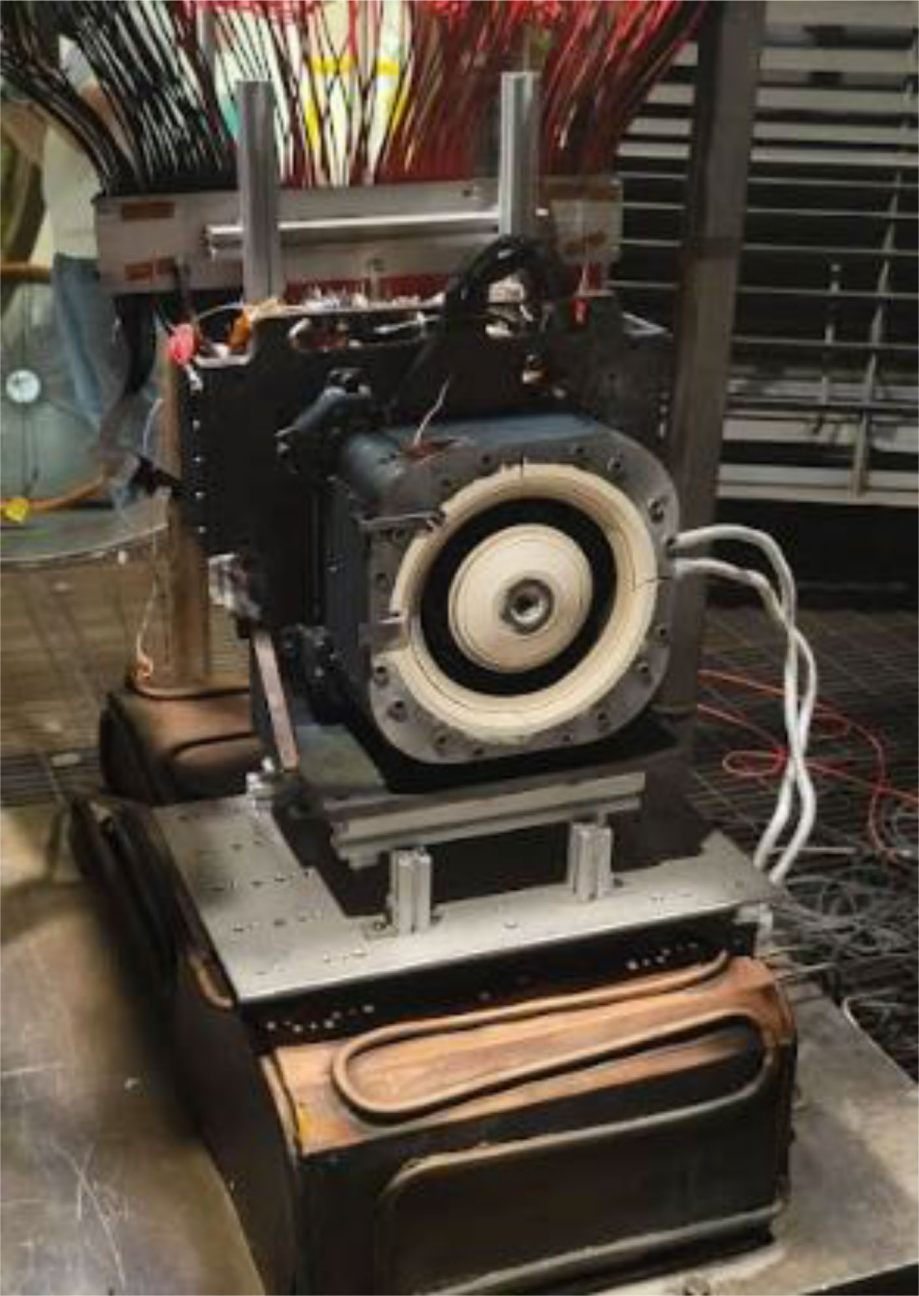
The BHT-7000 Hall effect thruster used for this work

The HET operated in this work is the Busek Hall Thruster 7000, BHT-7000, in a grounded thruster body electrical configuration. The BHT-7000 is based upon the evolution of the BHT-5000 thruster and is the latest design version in the moderate power class of HETs greater than 6 kW. Its predecessor, the BHT-6000, was selected by NASA to support Gateway’s Power & Propulsion Element and is currently undergoing flight qualification acceptance testing with a planned launch date in 2025 [9]. The BHT-7000 utilizes a center-mounted, 50-A barium-oxide cathode. The thruster offers breath in performance in both xenon and krypton making it an ideal candidate for this research effort. Figure 2 shows pictures of the BHT-7000 test article.

Two HET discharge operating conditions were examined to determine the effect of increased discharge current on the degree of electrical coupling with the facility. The first thruster operating condition was 4.5 kW at 300 V, 15 A. The 4.5-kW discharge condition was selected because it is currently the state-of-the-art for high-power HETs with flight heritage. Therefore, characterizing this operating condition will be a relevant contribution for practical applications in the commercial and government space propulsion sectors. The second discharge operating condition was 6 kW at 300 V, 20 A and was selected to measure the effect of increased discharge current levels coupling to the facility. The BHT-7000 was operated on krypton at the two discharge operating conditions shown in Table 1. The thruster discharge was powered using a Magna-Power TS800-54 50-kW DC power supply. An RC filter, comprised of a high-power 0.533-Ω resistor bank and 100 *µ*F-capacitor, is installed between the DC supply and the HET power lines to protect the supply from the reflected discharge current oscillations. The anode and cathode flow rates are maintained at their respective values using commercially available MKS GE50A mass flow meters. Mass flow calibration was performed at these two flow conditions using a Mesa Labs DryCal 800 to ensure output flow uncertainty is within 2% before and after the test campaign.

**Table 1:** BHT-7000 discharge operating conditions for this experiment

Discharge Power Operating Condition	V_dis_ (V)	I_dis, dc_ (A)	$\:\dot{m}$_anode_ (mg/s)	$\:\dot{m}$_cathode_ (mg/s)	I_mag_ (A)
4.5 kW	300 V	15 A	12.28	0.86	3
6 kW	300 V	20 A	15.39	1.08	3

### Test facility

The experiment was conducted at the Georgia Institute of Technology’s High-Power Electric Propulsion Laboratory in Vacuum Test Facility 2 (VTF-2). The facility is a stainless-steel cylinder with domed end caps measuring 4.9 m in diameter by 9.14 m in length. The chamber generates a high vacuum environment in two sequential stages. First, a Leybold RUVAC RA 5001 root blower backed by a single-stage, rotary vane SOGEVAC SV630 B mechanical pump brings the facility base pressure from atmospheric conditions to approximately 2.5 × 10^− 2^ Torr. Once the chamber outgas leak rate is less than 0.1 mTorr/min, the blower and mechanical pump assembly is turned off. Then, ten liquid nitrogen-cooled PHPK TM1200i cryopumps are activated, enabling the facility to achieve high-vacuum base pressures of less than 2 × 10^− 8^ Torr-N2 in approximately 24 h. Liquid nitrogen (LN2) is supplied to each cryopump in the temperature range between 90 and 110 K via vacuum-jacketed feed lines using a Stirling Cryogenics SPC-8 closed-loop, recirculating nitrogen liquefication system.

Three Varian 571 Bayard-Alpert hot filament ion gauges were used to measure the facility pressure at the chamber wall and 1-m away from the thruster exit plane. An Agilent XGS-600 controller was used to read out the ion gauge current measurements. The facility pressure readouts from the XGS-600 controller were collected digitally using LabView software at two data samples per second. The facility operational pressure measured by the internal gauge was maintained below 5.6 × 10^− 6^ Torr-Kr for the 4.5 kW, 15 A test condition and below 8.2 × 10^− 6^ Torr-Kr for the 6 kW, 20 A test condition. Moreover, the pressure was continuously monitored and logged for the entire duration of the experiment.

### Diagnostics

**Fig. 3:**
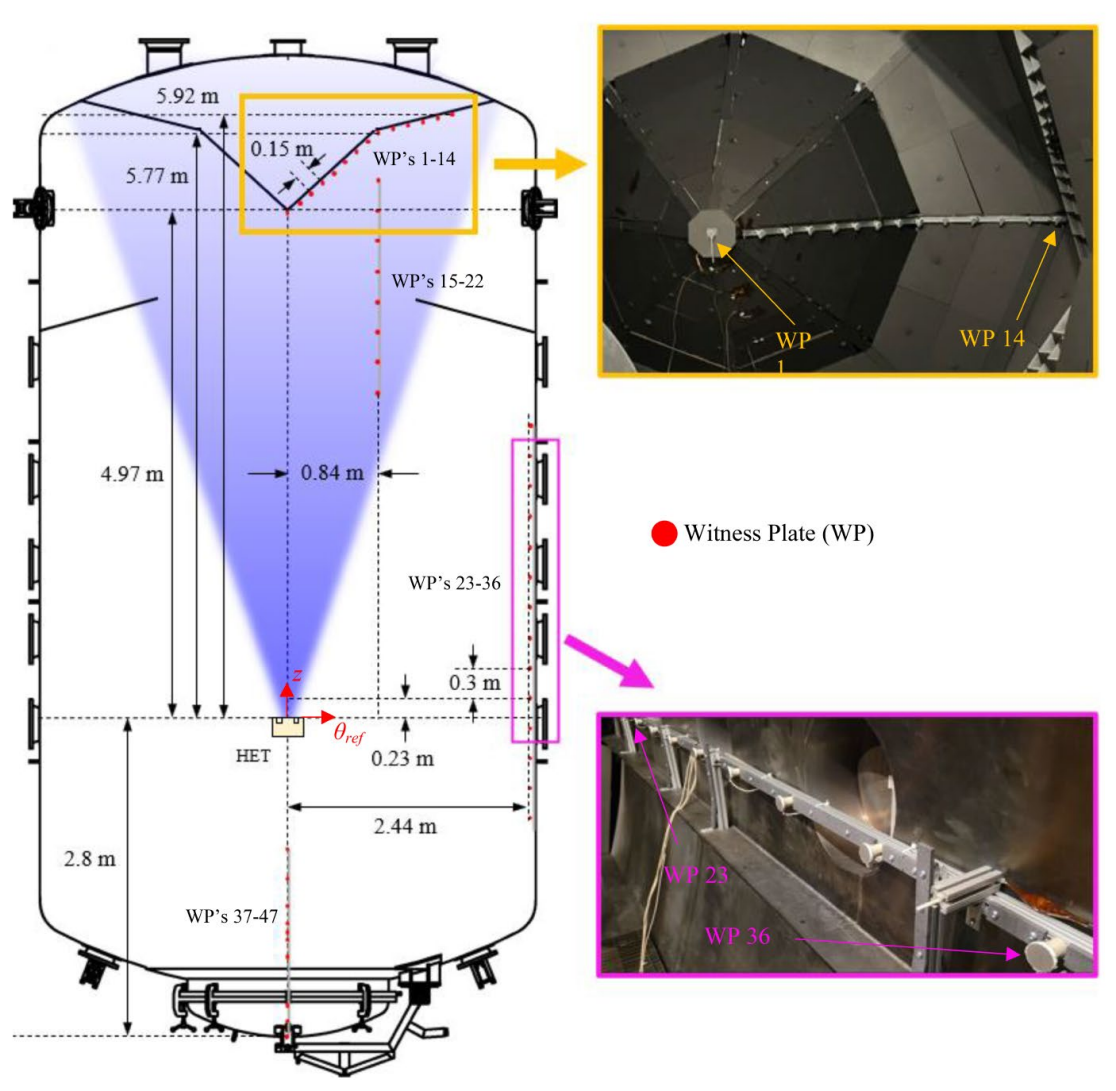
Schematic of the location of all 47 witness plates along VTF-2’s walls

An array consisting of 47 witness plates (WPs) was installed along the chamber walls to measure the local ion and electron current fluxes and floating potentials at different locations throughout the facility. To achieve this, each WP was installed as close as possible to, but electrically isolated from, the facility wall and connected to a sourcemeter to obtain local *I-V* characteristics at that location. This diagnostic measures the ion and electron current near the facility walls and quantifies the degree of electrical coupling between the HET plume and the facility. In addition, the WP array provides a spatial resolution of the beam and CEX ion current fluxes near the facility wall, making it the first measurement of its kind and a novel contribution for the computational research groups modeling electrical facility effects in EP testing. A schematic showing the location of all 47 WPs along the facility relative to the thruster is given in Fig. 3.

The spacing between each WP outside the ion beam was set to one thruster outer diameter of approximately 30 cm to capture the plasma physics on the order of the thruster exit plane length scale. For WPs inside the thruster plume, the spacing was based on the BHT-7000 radius for increased resolution of the plasma environment in this region. Thus, the spatial resolution of the plasma environment for this work is defined by the geometry of the HET. The 47 WPs were installed between the aft and fore end regions of the chamber to capture a complete 180° field-of-view between the thruster center and the facility walls. The WPs were mounted on 8020 extrusions with graphite shielding to reduce sputtering effects, as depicted in Fig. 3.

**Fig. 4:**
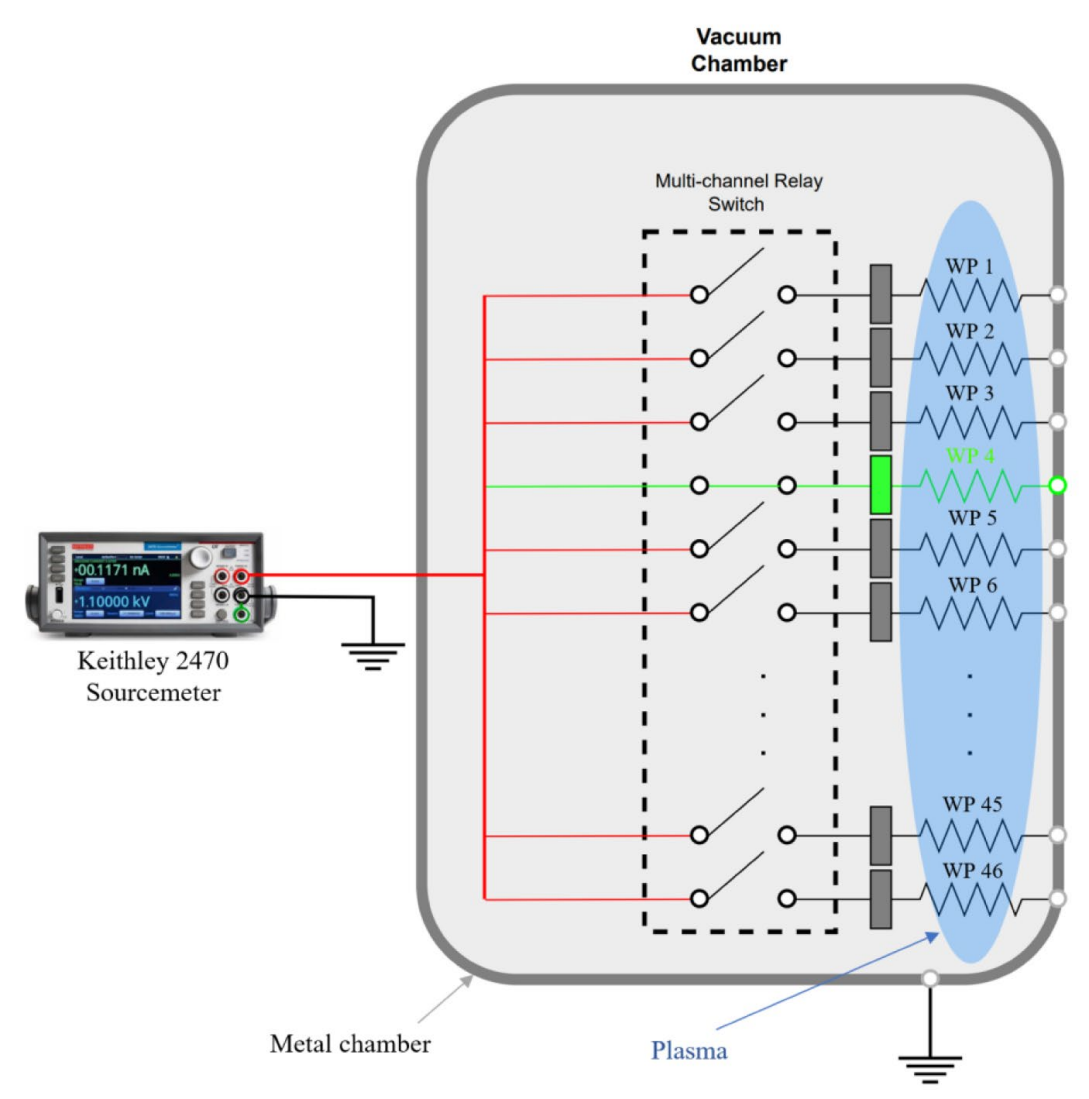
Relay switchboard circuit system to operate the 47-witness plate array

Each WP is a circular disk 5.08 cm in diameter made from 0.48-cm thick 304 stainless steel sheets and of the same facility wall metal. To minimize backsputtering, the plasma-facing surface of the WPs are coated with tungsten. All WPs were electrically isolated from nearby grounded structures using ceramic isolators and within 6 cm of the facility wall to measure the local ion and electron currents at that location. Furthermore, the sides of the disks are coated with Ceramabond 571 to ensure only the plasma-exposed surface collected current. A 48-channel, 3 A ProXR relay switch system by Relay Pros was used to activate individual WP circuits and sequence through the array to collect local *I-V* characteristics. A Keithley 2470 Sourcemeter was used to bias the individual witness plate between − 20 V and + 50 V, in steps of 0.1 V, with respect to facility ground, to detect ion and electron saturation regions. Each plate was scanned three times to obtain average *I-V* values and error statistics at that facility location. A schematic showing the electrical circuit when collecting *I-V* data for a witness plate using a multi-channel relay board is given in Fig. 4.

**Fig. 5:**
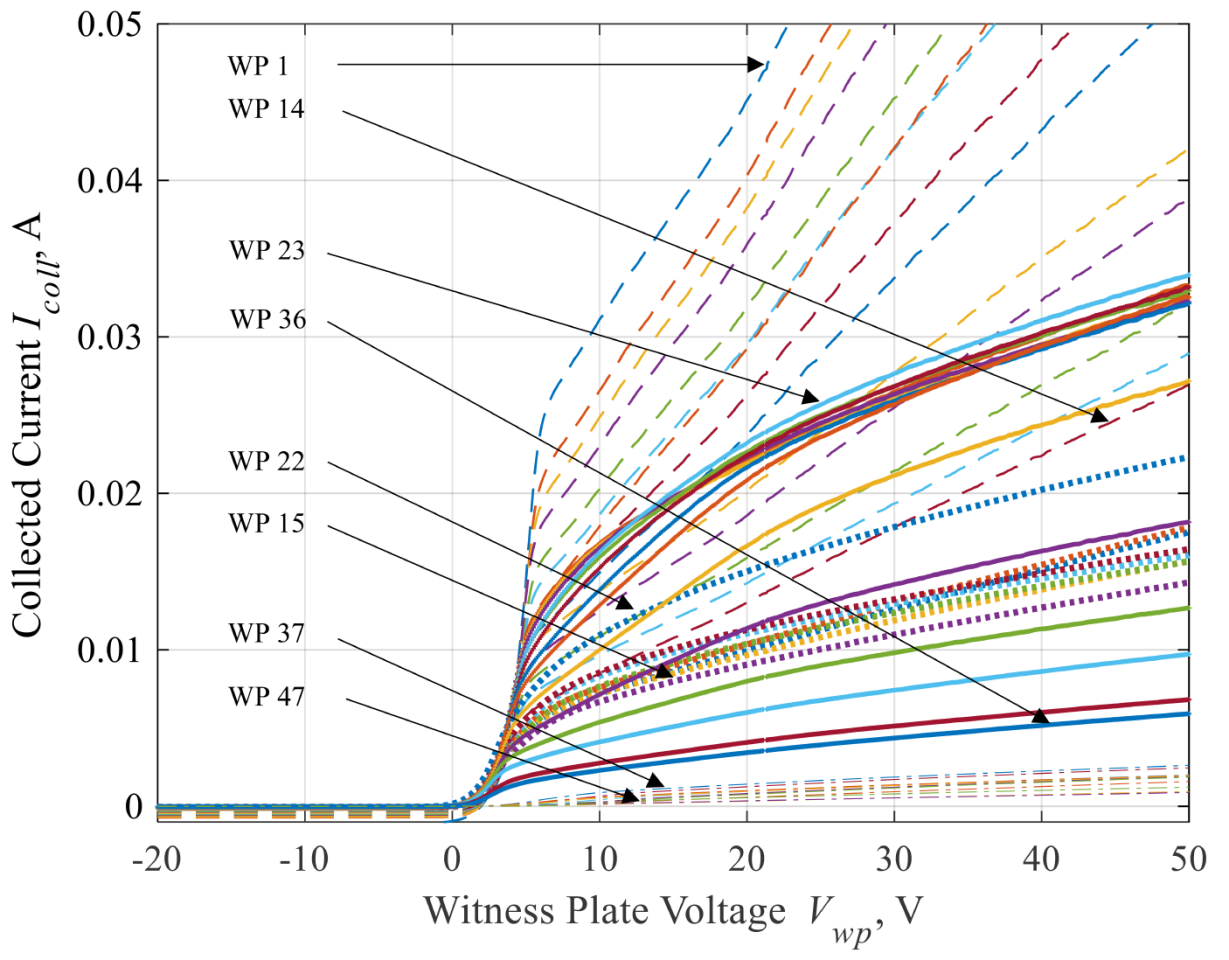
Sample trace of all 47 *I-V* curves for the BHT-7000 operating at the 6-kW discharge operating condition

In Fig. 5, we share a sample dataset showing all 47-witness plate *I-V* traces captured for the 6-kW test condition. The WPs are numbered 1 through 47, with WP 1 being the witness plate downstream of the HET centerline and WP 47 being the witness plate directly behind the thruster. The reader can see the evolution of each *I-V* trace as a function of the WP’s respective location throughout the facility. For example, the magnitude of electron current collected around the local *V*_*p*_ decreases as the interrogation point moves from inside the HET plume and into the background plasma.

The local ion saturation current, *I*_*i, sat*_, floating potential, *V*_*f*_, and electron saturation current, *I*_*e, sat*_, were obtained from each plate’s *I-V* trace. The *I*_*i, sat*_ was determined by taking the average of ion current values from − 20 V up to the local inflection point below *V*_*f*_. The *I*_*e, sat*_ was determined by taking the average of electron current values from the estimate for the local plasma potential up to 50 V. Finally, *V*_*f*_ was extracted from the net zero current condition in each *I-V* trace. The three measurements primarily indicated the quantity of ions or electrons fluxing locally at their respective near-facility wall locations.

We remark that our attempt to ensure each WP had a direct line-of-sight with the HET centerline was limited by facility support structures during certain test conditions. In particular, vacuum pumping surfaces and other facility diagnostics obstruct the line-of-sight of some of the witness plates with the thruster. Hence, data collected by WP’s 15–22 are not shown.

**Fig. 6:**
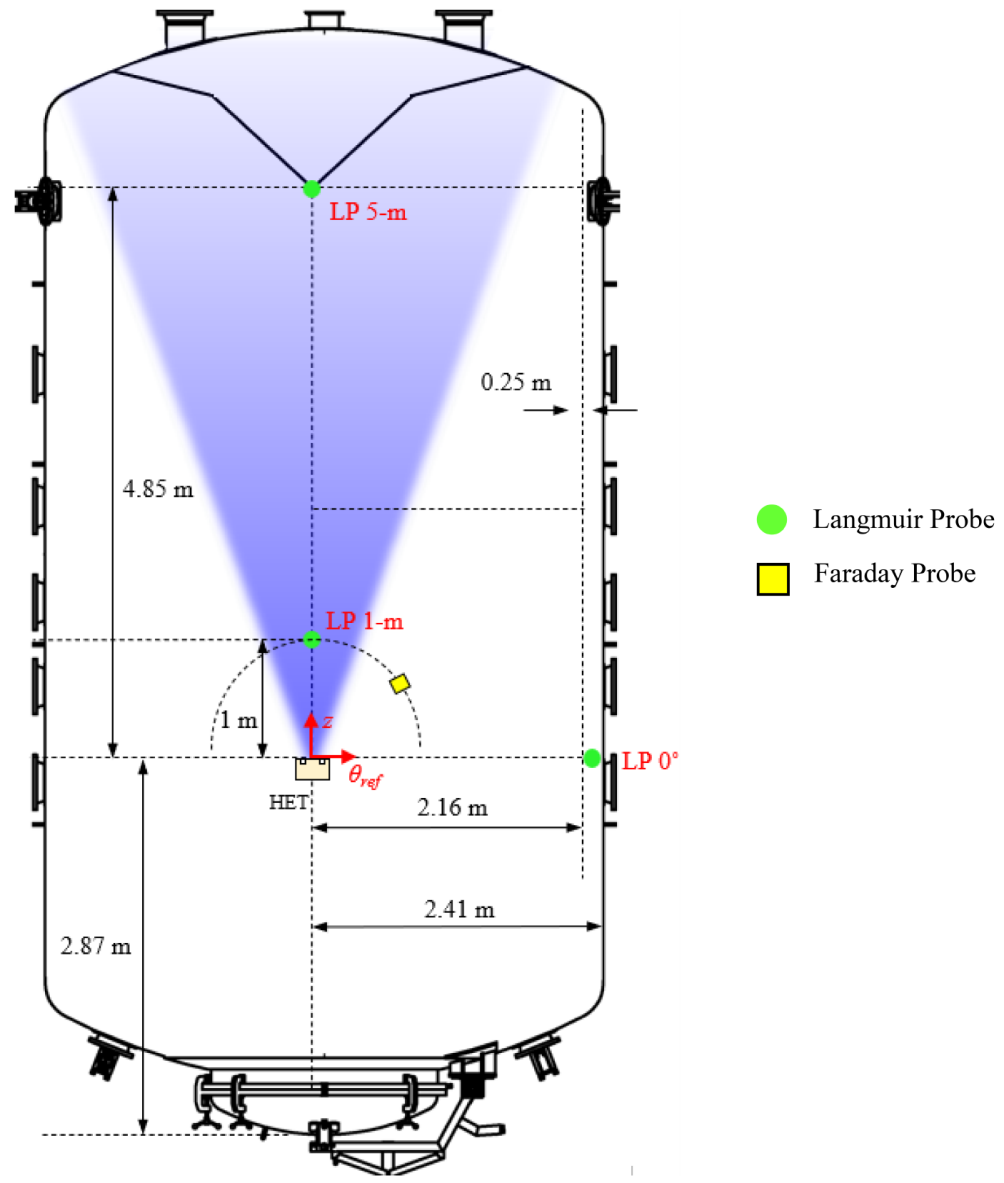
Location of the three Langmuir probes and Faraday probe placed inside VTF-2

In collaboration with Stanford’s Plasma Dynamics Modeling Laboratory, three locations throughout VTF-2 were identified for this experiment. The three locations with distances relative to the thruster exit plane in cylindrical coordinates are shown in Fig. 6. The purpose of interrogating at these locations was to characterize and compare the plasma properties within the HET plume and the background plasma environment. The probes designated “LP 1-m” and “LP 5-m” are all colinear with the HET centerline and immersed in the HET plume. Note that their labels are based on their approximate distances as shorthand with their actual distances provided in Fig. 6. The probe labelled “LP 0°” is 0.25 m offset from the facility wall. This probe was outside the main HET plume and characterized the background plasma region downstream of the HET exit plane at the sides.

For this work, a cylindrical-type Langmuir probe constructed from 0.13 mm diameter thoriated tungsten wire served as the electrode. The LP 1-m and LP 5-m probe lengths were 6.4 mm long and the LP 0˚ probe length was 12.7 mm, slightly longer given its location relative to the HET plume. The tungsten tip was housed inside a 6.4-mm diameter by 65-mm long alumina ceramic tube and oriented with the tip facing the thruster. A Keithley 2470 Sourcemeter was used to bias the probe from − 30 V to + 20 V, in steps of 0.5 V, with respect to ground and measure the collected current. Three probe sweeps are collected for each probe location for statistics. The data output from the Keithley 2470 was commanded and recorded in real-time using a LabView program to obtain the final local *I-V* characteristic curve.

A nude-type JPL design Faraday probe with a 2.31 cm diameter collector electrode was mounted on a rotary stage and swept in front of the thruster, tracing a 1-m radius semicircle with the thruster at the center [10]. The Faraday probe sweep trajectory relative to the thruster is shown in Fig. 6. The collector electrode has a tungsten coating to reduce secondary electron emission from its surface. The surrounding guard ring has a 2.54 cm outer diameter that is electrically isolated from the collector electrode using Macor ceramic spacers. The collector and guard ring are biased to -30 V with respect to ground using a Keithley 2470 Sourcemeter that also measures current on the collector electrode. The Faraday probe was swept using a Parker Daedal 200RT series rotary table with a positional accuracy of ± 0.17° at a slew rate of 2°/s. Similarly, three probe sweeps are collected at each thruster operating condition for statistics.

## Experimental results

This section provides all the results pertinent to quantifying aspects of the HET Plume-Facility current pathways. We start this section by reporting the ion and electron saturation currents measured by the 47 near-facility wall WPs. These measurements are then converted to ion current densities to estimate the total beam ion current and total CEX ion current. This was done to acquire estimates of the two additional current pathways found in ground-based test facilities across the two thruster operating conditions. The plasma properties determined from the Langmuir probe scans are presented as well. These results are used to determine the inductance due to plasma oscillations and plasma sheath capacitance, as discussed in section V.

**Fig. 7:**
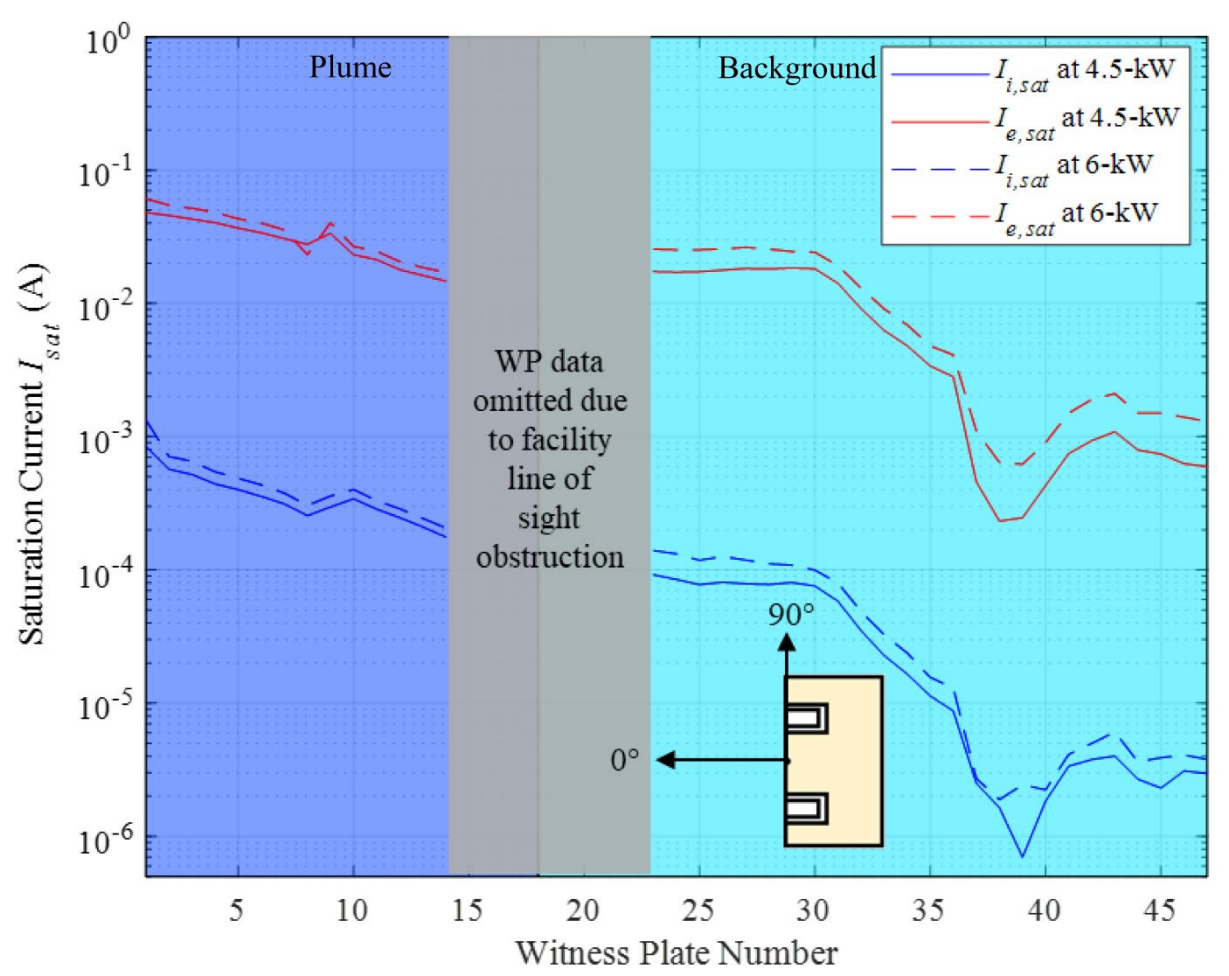
*I*_*i, sat*_ and *I*_*e, sat*_ profiles at the 47 near-wall locations for the 4.5 kW, 15 A and 6 kW, 20 A test conditions. Note: The thruster orientation shown here is with respect to the WPs, in accordance with Fig. 3, to depict which WPs lie in front of and behind the thruster exit plane

The ion saturation currents and electron saturation currents for the 4.5 kW, 15 A and 6 kW, 20 A discharge operating conditions are shown in Fig. 7. For the 4.5 kW, 15 A operating condition, the ion saturation current varies from 7.0 × 10^− 7^ A to 8.4 × 10^− 4^ A, and the electron saturation current varies from 2.3 × 10^− 4^ A to 4.8 × 10^− 2^ A. For the 6 kW, 20 A operating condition, the ion saturation current varies from 1.9 × 10^− 6^ A to 1.3 × 10^− 3^ A and the electron saturation current varies from 6.2 × 10^− 4^ A to 6.1 × 10^-2^A. As expected, more ions and electrons were detected at all WPs as the discharge current increased from 4.5 kW, 15 A to the 6 kW, 20 A operating condition. Note that WP’s 15–22 are omitted from this analysis as explained in section III.

The ion current density distribution measured by the Faraday probe scans provided an estimate of the ion beam current generated by the HET and served as a complementary diagnostic to the WP array. For the 4.5 kW, 15 A test condition, *I*_*i, beam*_ was 11.8 A with a *θ*_*div*_ of 25 °. For the 6 kW, 20 A test condition, *I*_*i, beam*_ was 15.7 A with a *θ*_*div*_ of 25 °. The beam divergence half-angle allows us to decompose the plasma environment into the plume and background plasma regions. For the *θ*_*div*_ of 25 ° and the layout of WPs in Fig. 3, WPs 1–14 are in the plume region and assumed to be pure beam ions whereas WPs 23–47 are in the background plasma region and assumed to be purely CEX ions. The effect of these assumptions is further addressed in the estimation below.

The ion saturation current measured in each region can be used to estimate the total beam and CEX ion currents fluxing to the chamber wall. To do this, each ion saturation current was transformed into a current density measurement and assigned a fraction of the total chamber surface area. The surface area of WP 1 is its own surface area. The surface areas for WPs 2–14 are 21.2 cm thick rings concentric with WP 1. The ring thickness for WPs 2–14 takes in account the beam dump geometry that has a 45˚ inclination. The surface areas for WPs 23–36 are 30 cm thick rings with a radius equivalent to the chamber radius. The contribution of WPs 37–47 on the total CEX ion current was determined by the average current density in that region and the total chamber area from WP 37 to the chamber door. For the 4.5 kW, 15 A test condition, these approximate surface areas provided a total ion current of 8.2 A contained in the plume and approximately 1.9 A of CEX ion current contained in the background plasma region. The sum of these two currents is roughly 86% of the ion beam current determined from the ion current density distribution from Faraday probe scans. For the 6 kW, 20 A operating condition, the estimated total ion beam current was about 9.7 A, and the total CEX ion current was about 2.7 A. The sum of these two currents is roughly 79% of the ion beam current determined from the Faraday probe scan. Differences between these ion current estimates to the measured *I*_*i, beam*_ from the Faraday probe are attributed to the choice in chamber surface areas when approximating ion current densities, low spatial resolution between WPs, and attenuation of current measurements due to line-of-sight obstruction for some of the WPs. We note that our assumption regarding the type of ions present in the plume and background plasma regions ultimately underestimates the CEX ion current fluxing to the chamber walls. This is because CEX ions are also present within the plume region and not accounted for in this analysis. Regardless, we believe these two ion currents are important to distinguish and estimate as these calculations are the first of their kind. Lastly, the two ion current estimates provided here correspond to the second and third current pathways in the HET Plume-Facility model in Fig. 1.

The inductance generated by the oscillating plume in the HET Plume-Facility model, as well as the plasma sheath capacitance, are dependent on local plasma properties. The electron temperature, electron number density, and plasma potential for the 4.5 kW and 6 kW thruster operating condition at the various Langmuir probe locations are shown in Tables 2 and 3, respectively. The uncertainty associated with each parameter was determined using the methods provided in [7]. We used the properties measured at LP 1-m to estimate *L*_*e*_ and the properties measured at LP 5-m and LP 0˚ to estimate *C*_*sh*_. Moreover, we used a wetted facility area of 293 m^2^ for both sheath interfaces and used the thruster’s outer diameter cross-sectional area of 0.07 m^2^ for inductance calculations. The inductance due to plume oscillations is 101.4 nH at the 4.5-kW test condition and 76.5 nH at the 6-kW test condition using the *l*_*eff*_ and *A*_*eff*_ given in section III. At the 4.5 kW, 15 A operating condition, the capacitance due to the plasma-wall sheath is 1.15 *µ*F in the plume region and 0.45 *µ*F in the background plasma region. At the 6-kW thruster operating condition, the capacitance due to the plasma-wall sheath is 1.79 *µ*F in the plume region and 0.66 *µ*F in the background plasma region.

**Table 2:** Plasma properties at three locations for the 4.5 kW, 15 A test condition

Probe Location	*T*_e_, eV	*n*_e_, m^− 3^	V_*p*_, V
LP 1-m	2.32 ± 0.36	2.97 × 10^16^ ± 1.23 × 10^15^	12.75 ± 0.25
LP 5-m	1.14 ± 0.17	4.61 × 10^14^ ± 2.00 × 10^13^	4 ± 0.13
LP 0°	1.88 ± 0.24	1.09 × 10^14^ ± 3.55 × 10^13^	6.24 ± 0.25

**Table 3:** Plasma properties at three locations for the 6 kW, 20 A test condition

Probe Location	*T*_e_, eV	*n*_e_, m^− 3^	V_*p*_, V
LP 1-m	2.24 ± 0.40	3.94 × 10^16^ ± 2.59 × 10^15^	12.5 ± 0.25
LP 5-m	1.13 ± 0.17	1.45 × 10^15^ ± 5.71 × 10^13^	4.75 ± 0.13
LP 0°	1.05 ± 0.18	2.24 × 10^14^ ± 1.26 × 10^13^	5.08 ± 0.13

## Discussion

This section provides insight on the HET Plume-Facility current pathways model and results to understand the degree of electrical coupling that exists between the HET plume and metallic chamber walls. The two dominant neutralization current pathways in ground-based facilities postulated in section V are supported by the experimental data presented in the article. In addition, each neutralization current pathway is quantified as percentages of the total ion beam current. The reactances present in the two distinct regions of the plasma environment are compared to each other. Moreover, the limitations of the model are reviewed.

### Impact of metallic chamber walls on neutralization current pathways

The model shows that the high electrical conductivity of the metal chamber walls assists in the neutralization of the two additional current pathways observed inside ground-based test facilities. The first neutralization pathway of the HET Plume-Facility model segment in Fig. 1 represents the recombination of cathode electrons with either beam or CEX ions. As established in [1], the recombination of ions with cathode electrons with *T*_*e*_’s of about 1.5 eV requires thousands of meters and instead will favor recombination with the metal chamber wall’s free electrons. Since recombination in the plume is unlikely, we can regard the first neutralization pathway in the model as having a much higher effective resistance compared to the electrical conductivity of the chamber wall body. Thus, the model appropriately captures that the dominant neutralization pathway for both ion current populations occurs through the metal chamber walls.

The nonnegligible ion and electron saturation currents measured by the 47 near-facility WPs provide direct evidence that the two additional current pathways identified in the model must be the dominant neutralization pathways in ground-based test facilities. That is, the recombination of beam and CEX ions with the metal chamber wall free electrons are the primary neutralization pathways in ground-based testing facilities. For the 4.5 kW, 15 A test condition, 8.2 A of ion current contained in the HET plume and 1.9 A of CEX ion current ion the background plasma electrically interact with the metallic vacuum chamber surfaces. For the 6 kW, 20 A test condition, 9.7 A of ion current contained in the HET plume and 2.7 A of CEX ion current in the background plasma electrically interact with the metallic vacuum chamber surfaces. As expected, both beam and CEX ion currents interacting with the test facility increased as the discharge current increased. In particular, CEX ion current increased approximately 20% in going from 4.5 kW, 15 A to 6 kW, 20 A test conditions. These conservative estimates show that the metal vacuum chamber must be conducting the equivalent charge through its body to neutralize the distinct ion populations. Furthermore, the role the vacuum test facility plays in neutralization increases proportionally to the discharge current. In addition, the conduction of charge through the chamber body must be spatially non-uniform and proportional to the current profiles shown in Fig. 7.

Electron current was also detected at all 47 WPs and roughly two orders of magnitude larger than the ion currents measured. This observation also shows that the local plasma electrons must be fluxing to the facility walls and participating in maintaining a net charge neutral boundary condition. However, assigning electron current to the two neutralization pathways provided by a test facility was not done because they are highly mobile and assumed to be Maxwellian.

The model also reveals a direct link between the current pathways related to ionization inside the discharge channel and the pathways related to neutralization in the plume. Imposing the net charge neutral boundary condition across the HET plume-facility segment requires that the sum of all the charge sinks at the recombination sites be exactly balanced by the charge source due to ionization. Thus, if the ionization rate inside the HET is fixed and mainly governed by the user-defined operating parameters, then plume neutralization processes downstream are also influenced by the same operating parameters. The dependency of plume neutralization on *I*_*i, beam*_, *R*_*coupling*_, *Z*_*dis, ch*_ upstream and the conductivity of the test facility walls downstream is more likely a stability concern for high-power HETs since the quantity of both beam and CEX ion current populations fluxing to the walls increase.

### Capacitive and inductive effects introduced by vacuum test facilities

The capacitance of the plasma-wall sheath is the main complex impedance element in the HET Plume-Facility pathways segment with reactances that effectively alter ion and electron current fluxes to the chamber wall. The model shows that the sheath capacitors for the background plasma, plume plasma, and electron-wall plasma regions all interact to satisfy the net charge neutral condition. If certain current pathways are more prevalent than others throughout the facility, the associated plasma-wall capacitor can influence the remaining current pathways. For example, as the facility pressure increases, so will the sheath capacitance for the background plasma region. As a result, ion-electron current pathway fluctuations may be attenuated and absorbed by the background plasma sheath capacitor, potentially concealing related *I*_*dis*_ dynamic characteristics.

We propose modeling the plasma-wall sheath interfaces as a parallel RC circuit to quantify capacitive effects on the discharge current’s amplitude and phase. In a parallel RC circuit, the current’s magnitude and phase increases as the capacitance increases. This is because the increase in capacitance decreases the total effective impedance of the parallel RC circuit which results in the increase in the current’s magnitude observed. From Eq. 4, the capacitance of the plasma sheath is primarily a function of *n*_*e*_ and *V*_*p*_ since dependence on *T*_*e*_ is relatively weak provided the data in Tables 2 and 3. Further inspection shows that sheath capacitance most notably increases proportional to *n*_*e*_^1/2^. In addition to this, the consideration of multiple RC circuit branches in parallel, as depicted in Fig. 1, has the effect of further reducing the equivalent impedance of the network. For example, alternative thruster body electrical configurations would provide new current pathways that are not readily captured by Fig. 1. Each of these additional plasma-surface interfaces would participate in the aggregate parallel RC circuit network to maintain a net charge neutral environment. Therefore, the increase in plasma number density and the addition of current pathways to ground lead to a capacitive effect that allows more charge to be stored in the sheath ultimately attenuating HET plasma oscillations. As an exercise for interested readers, we provide an analysis of a simple parallel RC circuit in the frequency domain to help illustrate the impact of capacitance on the current’s amplitude and phase in Appendix A.

The time-varying plasma oscillations present in the HET discharge cause an effective inductance with estimated values provided in section IV. Since the geometric parameters are the same for both test conditions, the inductance was found to be most sensitive to the measured plasma number density. The number density between the 4.5-kW and the 6-kW test conditions increased by 30%, consequently reducing the plume inductance proportionally based on Eq. 2. The plasma properties at the LP 1-m location for both test conditions do not show a large disparity and therefore experience similar inductive effects. However, the plasma plume densities observed in the space operational environment, although unknown, can be reasonably presumed to be lower than what is measured in ground-based test facilities. Employing a conservative estimate in which the *n*_*e*_’s presented in this work are divided by ten shows that the effective inductance can be on the order of 1000’s of nH’s. Our point here is that the without precise measurement of the plasma plume properties in the thruster’s operational environment, the potential effects of plume inductance, as presented in this paper, remains an area to be explored.

### Limitations of the HET plume-facility model

Converting the current pathways model into an equivalent HET discharge-facility electrical circuit has many limitations. There are three main challenges we must address in this transition. The first main challenge is that the plasma environment inside vacuum chambers is 3D and inhomogeneous, with discernible plasma properties inside and outside the emitted thruster plume. The ion beam is characterized by an ion velocity distribution function in energy and direction as well as volumetric expansion downstream. Additionally, there is a slower-moving CEX ion population emanating from the plume that randomly fluxes to all parts of the facility and exhibits different plasma properties than the highly energetic plume plasma. Furthermore, the 3D shape of the vacuum chamber with respect to the location of the thruster results in varying distances, and therefore, the chamber wall shares its conduction electrons for recombination events at different rates depending on the local influx of ions. In contrast, electrical circuits require that electromagnetic phenomena be spatially confined to discrete bodies like resistors, capacitors, and inductors. A second impediment is that the plasma consists of multiple charge species, at a minimum singly-charged ions and electrons, instead of only electrons as tracked in typical electrical circuits. Third, the plasma environment contains charge sources, ionization, and charge sinks, recombination, that are time-dependent physical processes and do not provide a closed path for charges as fundamentally required by electrical circuits. Thus, we had to deviate from classical electrical engineering practices while adhering to charge and energy conservation equations to develop the model.

## Conclusion

Electrical facility effects in EP characterizes the effects electrically-conductive vacuum chambers have on the performance and stability of HETs. In a previously published companion paper, a current pathways model was developed that represents the electrical coupling between a HET and the ground-based vacuum test facility operational environment. The model shows that vacuum chambers provide two additional pathways related to HET plume neutralization that are absent in the space operational environment: (1) CEX ion currents neutralizing with side and rear facility surfaces and (2) beam ions neutralizing with downstream facility surfaces. In this work, we quantify aspects of the current pathways model to characterize the role metal vacuum chambers play in the thruster’s discharge circuit as a function of increasing discharge current.

Experimental results from operating a 7-kW class HET at 4.5 kW, 15 A and 6 kW, 20 A on krypton provide direct evidence that the HET plume electrically interacts with the test facility’s metallic walls to satisfy charge neutrality. Ion and electrons currents were detected and measured at 47 near-facility wall locations at the two discharge operating conditions confirming that recombination of ions with free electrons supplied by the wall is the dominant neutralization pathway. In addition, Langmuir probe data was used to quantify the inductance due to plasma oscillations as well as the capacitance of the sheath between the plasma environment and metal chamber wall. The measured inductance and capacitance indicate that the dynamic characteristics of the HET discharge may be attenuated by ground-based vacuum test facilities. For example, an increase in plasma-wall sheath capacitance may help conceal a HET’s AC characteristics in test facilities. Additionally, lower plasma plume number densities observed in the space operational environment may affect the inductive behavior of HETs. The current pathways model is a step forward in understanding and quantifying the electrical coupling between the overall HET plume and its local operational environment and the corresponding effects on a thruster’s dynamic characteristics.

## Figures and Tables

**Fig. 8:**
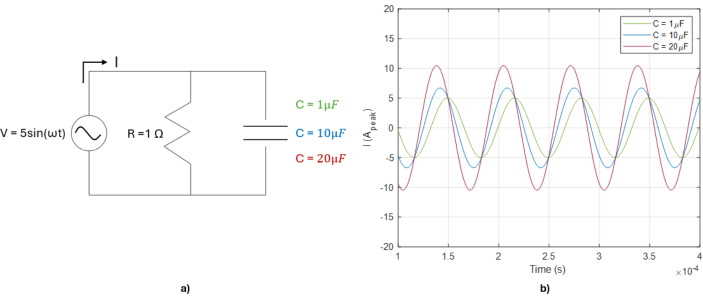
Parallel RC circuit analysis: (**a**) electrical circuit (**b**) current as a function of time

**Fig. 9:**
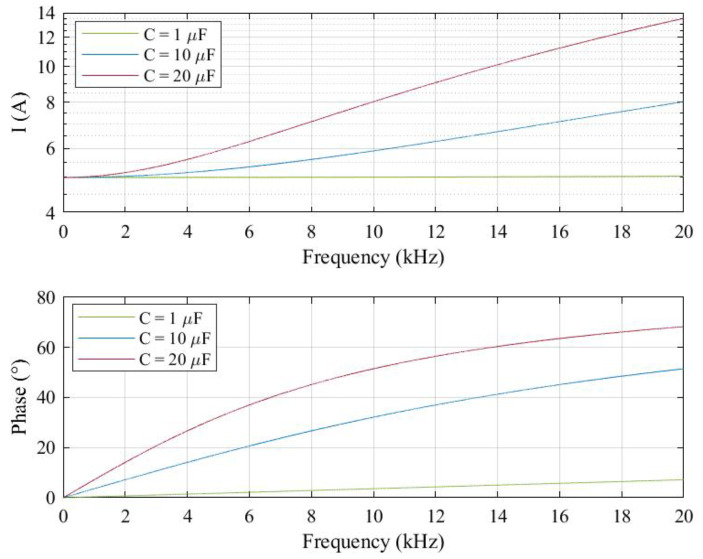
Current magnitude and phase as a function of frequency

## Data Availability

No datasets were generated or analysed during the current study.

## Appendix A

For this exercise, the AC voltage signal is applied to the RC load for the three capacitor values shown in Fig. 8a. The AC voltage is fixed at an amplitude of 5 V based on the average *V*_*p*_ measured by the Langmuir probes near the chamber wall given in Tables 2 and 3. Additionally, a plasma sheath resistance of 1 Ohm was assumed for simplicity. The capacitor values are 1, 10, and 20 µF in order to quantify the effect of increasing sheath capacitance on the total current through the circuit. Figure 8b shows the resulting current response as a function of time for 15 kHz – a typical breathing mode frequency. Figure 9 presents the magnitude and phase of current as the capacitance increased across the frequency range of 1 to 20 kHz. As both capacitance and frequency increase, more current is allowed to flow through the RC circuit with an associated phase shift.

## Nomenclature

A_sh_: Sheath surface area, m^2^
A_eff_: Effective area, m^2^
C_sh_: Sheath capacitance, F
e: Elementary charge, 1.60218 × 10^-19^ C
I_dis_: Discharge current, A
I_i, beam_: Beam ion current, A
I_i, CEX_: Charge-exchange ion current, A
I_e, beam_: Electron current for ion beam neutralization, A
I_e, CEX_: Electron current for CEX ion neutralization, A
I_e, ionization_: Electron current for ionization, A
I_e, plume_: Electron current for plume neutralization, A
I_e, rec_: Electron current for recombination in the plume, A
I_e, wall_: Electron current to the chamber wall, A
k_b_: Boltzmann constant, 1.38065 × 10^–23^ J/K
L: Inductance, H
l_eff_: Effective length, m
m_k_: Mass per particle of the k^th^ species, kg
n_k_: Number density of the k^th^ species, cm^-3^
P_op_: Facility operational pressure, Torr
Q: Collision cross-section, m^2^
R_sh_: Sheath resistance, Ω
T_e_: Electron temperature, eV
T_w_: Chamber wall temperature, eV
V_dis_: Discharge voltage, V
V_p_: Plasma potential, V
V_w_: Chamber wall potential, V
<v_i_ >: Mean ion velocity, m/s
Z_dis, ch_: Effective impedance of the discharge channel, Ω
Z_eff_: Effective ion charge state
ε_o_: Permittivity of free space, 8.8541e × 10^–12^ F/m
θ_div_: Ion beam divergence half-angle, ˚
λ_D_: Debye length, m
">ν_k_: Collision frequency of the k^th^ charge species, s^-1^
σ_e_: Electron conductivity, Ω^-1^
